# Survival Analysis of Clinical Cases of Caseous Lymphadenitis of Goats in North Shoa, Ethiopia

**DOI:** 10.1155/2020/8822997

**Published:** 2020-08-17

**Authors:** Erdachew Yitagesu, Enyiew Alemnew, Abebe Olani, Tadiwos Asfaw, Chekol Demis

**Affiliations:** ^1^Debre Birhan Agricultural Research Center, P.O. Box 112, Debre Birhan, Ethiopia; ^2^Department of Parasitology, National Animal Health Diagnostic and Investigation Center (NAHDIC), P.O. Box 04, Sebeta, Ethiopia

## Abstract

Caseous lymphadenitis (CLA) is a bacterial disease of small ruminants causing abscesses in lymph nodes of the body and internal organs. A longitudinal study from 2011 to 2019 was undertaken at Ataye site of Debre Birhan Research Center to estimate the prevalence and incidence, identify associated risk factors, and characterize the causative bacteria. 1025 goats were followed up for the CLA study. Survival analysis was done using SAS 9.4 software program. Biochemical tests and Biolog microbial identification system were used to characterize the bacteria. In the recurrent survival data analysis, there were 1,138 clinical observations and 214 CLA cases (18.8% prevalence) based on clinical diagnosis. The incidence rate was 0.14 cases per animal year. From a total of 214 cases, 130 have occurred once and 84 cases are recurrent cases following first cases. The cumulative failure rates were 68% for newborn and 64% for yearling age groups. The parotid lymph node was the most frequently affected site. Proportional hazard analysis results showed that sex, age, and breed were associated (*p* < 0.05) with CLA incidence. Females, newborn age group, Boer, and Boer × Central Highland Goat (CHG) were at higher risks compared to males, yearling age group, and CHG goats, respectively. The present study indicated that a high incidence rate of CLA in the goat farm is difficult to control and prevent because of its poor response to clinical treatment. Thus, control and prevention should focus on the spread of the disease such as isolation of clinically sick animals, culling, and vaccination of flocks.

## 1. Introduction

Abscess disease, commonly known as Morel's disease and caseous lymphadenitis (CLA), is a bacterial disease of sheep and goat causing abscesses in lymph nodes of the body and internal organs, especially the lung. It is a contagious disease distributed worldwide and lacks effective control measures. Once introduced into a sheep or goat flock, it is very difficult to control because of its poor response to treatment, its ability to persist in the environment, and the limitations in detecting subclinically infected animals [1, 2]. CLA is a disease caused by *Corynebacterium pseudotuberculosis* bacteria. It is commonly found in animals, such as sheep, goat, and cattle, from where the infection is transmitted to humans. These bacteria are Gram-positive, nonmotile pleomorphic rods (about 0.5 *μ*m in width) that often present a characteristic Chinese letter or palisade arrangement in the smear. *C. pseudotuberculosis* is a mycolic acid-containing facultative intracellular parasite that multiplies inside the macrophage. Its natural habitat is skin, mucus membranes, and gastrointestinal tract of normal sheep and soil of sheep pens [3, 4]. Accurate CLA diagnosis is based primarily on clinical observations (external abscesses) [5] and the identification of *C. pseudotuberculosis* by phenotypic and biochemical tests; this is important to differentiate this bacterium from other abscess inducing pathogenic agents, such as *Arcanobacterium pyogenes* or *Pasteurella multocida* [2, 6, 7].

It causes considerable economic losses to sheep and goat producers as it reduces milk yield and meat and wool production. Also, it decreases reproductive efficiencies. It is the main cause of condemnation of sheep carcasses in slaughterhouses in Australia, one of the world's largest producers of meat and wool [8, 9]. Unfortunately, it is the internal abscesses that are fatal, whereas external abscesses are generally responsible for disease transmission [1]. In Ethiopia, recent findings indicate that 15% prevalence of caseous lymphadenitis was reported from goats slaughtered in Luna Export Abattoir originated from district area of Borena Range Land [10] and 10% prevalence of local abscess was also reported in Boer goats of Adami Tulu Agricultural Research Center Nucleus site being the major disease next to GIT parasites, keratoconjunctivitis, ticks, and respiratory problems [11]. 4.7% prevalence of caseous lymphadenitis was also reported from the Boer goat breeding station of Southern Ethiopia [12]. In Ethiopia, the disease was not yet characterized at farm level. Therefore, the objective of this study is to estimate the prevalence, incidence, and risk factors of caseous lymphadenitis and to identify the causative bacteria.

## 2. Materials and Methods

### 2.1. Study Area and Animals

**Figure 1:**
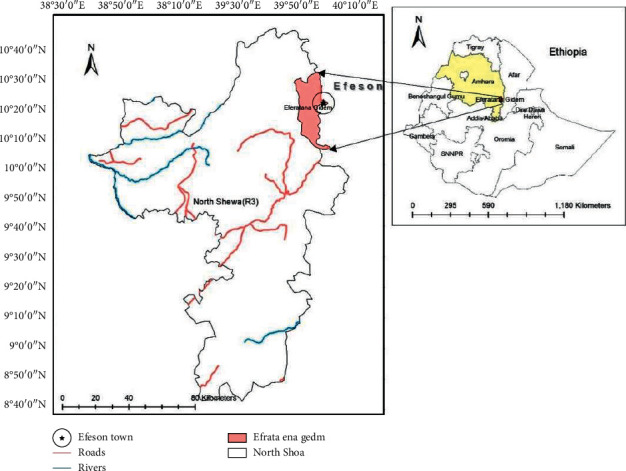
Map of Ethiopia showing the region, zone, district, and town where the study site is located.

The study was conducted at the on-station Boer *× * Central Highland Goat cross-breeding program carried out at Ataye Research site, Debre Birhan Agricultural Research Center, Ethiopia. The site is located in central Ethiopia and the climate is characterized by bimodal rainfall consisting of the long rainy season (June-September), short rainy season (February-May), and dry season (October-January) [13]. The site's geographic coordinate reference is 10°35′ N latitude and 39°93′ E longitude and is located at 1491 m above sea level altitude (Figure 1).

The study animals were a mix of different goat breed groups including Boer, Boer cross with Central Highland Goat, and Central Highland Goats. The flock was managed semi-intensively with grazing and supplement. The supplement includes ad libitum grass hay, chopped pasture (Napier grass, *Desmodium* spp., and vetch), and concentrated supplement based on their body weight. Detail summary of breeding, feeding, management system, and data recording of the flock are presented in [14, 15].

### 2.2. Clinical Examination and Sample Collection

The study was carried out on a goat farm maintained under a semi-intensive system in the lowland of North Shoa, Ataye District, Ethiopia. The study was conducted over a period of 8 years (June 2011 to January 2019) using a longitudinal (both retrospective and prospective) follow-up study design. A total of 1025 goats of breeds, 287 Boer goat, 515 Boer × CHG, and 223 CHG), were followed up and clinically examined for the presence of enlarged and abscessed superficial lymph nodes and internal caseous abscess during postmortem examination. Pus samples from 32 goats with lesions suspected of caseous lymphadenitis were taken after shaved and disinfected with 70% alcohol or savlon, and then, an incision was made with a sterile blade and the samples were transported in icebox to the microbiology laboratory of Debre Birhan Agricultural Research Center.

### 2.3. Descriptive Statistics and Survival Analysis

Data collected from clinical cases and laboratory tests were entered into an Excel spreadsheet. Clinical records were rearranged as time-to-event data form in the Excel spreadsheet. The starting point of follow-up was the date (day/month/year format) that the goat joined the farm through birth, purchase, or transfer from other research centers. The event/failure time was the date (day/month/year format) a goat shows CLA clinical sign.Since the disease is characterized by a recurrent relapse of abscess due to poor response to antibiotic treatment, the data were rearranged according to the counting process data layout with multiple observations per subject [16]. Goats that fail in the first time will be again recorded using the first failure time as the initial time. Censored observations are goats that leave out the farm by death (the most common one), by transfer, or end of the study period.

The estimation of the survivor function was computed as follows [17]:

(1) $$\begin{array}{c} \;\hat{S}\left(t\right)\;=\prod_{j:\;t_{j}\leq t}\left(1-\frac{d_{j}}{n_{j}}\right), \end{array}$$

where *Ŝ* (*t*) is the value of survival function at a time *t*_*j*_, *n*_*j*_ is the number of goats without caseous lymphadenitis cases at time *t*_*j*_, and *d*_*j*_ is the number of goats with caseous lymphadenitis cases at time *t*_*j*_. Survival curves were constructed with the Kaplan–Meier method, and we used the survival curve plotting macro %NEWSURV [18].

The risk factors associated with caseous lymphadenitis were assessed using multivariate Cox regression analysis adjusted for sex, age, breed, season, and year. Statistical analyses were performed using SAS statistical software version 9.4 [19]. We used the PROC PHREG option called COVSANDWICH that corrects for the dependence of repeated events on the same goat over time. This option invokes a method variously known as the robust variance estimator or the modified sandwich estimator, developed for Cox regression by Allison [16] and Lin and Wei [20]. The proportional hazard assumption of covariates was checked using PH ASSES option, and for the violated variables, we add time interaction in the model. Year when the goat joined the farm was used as a stratifying variable. *p* values <0.05 were considered as statistically significant. Then, Weibull proportional hazard model for the caseous lymphadenitis case of a particular goat at a time (*t*) was designed as follows:

(2) $$\begin{array}{c} \lambda \left(t\right)=\lambda_{0\;}\left(t\right)*\;\exp\left({\text{Age}}_{i\;}+{\text{Sex}}_{j}+{\text{ Season}}_{k}\right), \end{array}$$

where *λ* (*t*) is the risk of caseous lymphadenitis or probability of goats being caseous lymphadenitis positive at time *t*, *λ*_0_ (*t*) is the baseline hazard function with shape parameter *p* and scale parameter *λ* of the Weibull distribution or *λ*_0_ (*t*) = *λρ* (*λt*)^*p*−1^, Age_i_ is fixed effect of the *i*^th^ age of goat when joined the farm, Sex_*j*_ is fixed effect of the *j*^th^ sex of goat, and Season_*k*_ is fixed effect of the *k*^th^ season of the year when the goat joined the farm.

### 2.4. Bacteriological and Biolog MicroStation with GEN III Microplate System Bacteria Rapid Identification

Thirty-two pus samples taken from suspected caseous lymphadenitis abscess were cultured on 7% sheep blood agar for 48–72 h at 37°C, and bacterial colonies were identified on the basis of morphological characteristics, biochemical tests, i.e., catalase, urease, trehalose, xylose, maltose, and glucose fermentation tests, and reverse CAMP test (antagonistic haemolysis between *Corynebacterium pseudotuberculosis* and *Staphylococcus aureus*) for phospholipase D (PLD) production [4]. Catalase, PLD, and urease-positive and nitrate-negative cultures were considered positive for *C. pseudotuberculosis*.

Out of 32 bacterial cultures, 12 samples of well-grown bacterial colonies on blood agar were transported to the National Animal Health Diagnostic Center, Sebeta, in icebox for further bacterial species identification. OmniLog (fully automated coated microplate-based bacterial identification system), that is, GEN III microplate with protocol A method, was used to test suspected colonies. A single colony grown on Biolog Universal Growth (BUG) agar medium was selected and emulsified into “inoculating fluid A” (IF A). According to the manufacturer's instructions, cell density of the bacterial inoculum was measured for a specified transmittance (90 to 98%) using a turbid meter, as specified in the user guide. For each isolate, 100 *μ*l of the cell suspension was inoculated into each of the 96-well coated microplate, using an automatic multichannel pipette, and incubated aerobically at 33°C for 22 h. The OmniLog identification system automatically reads each microplate and provides identification called species/subspecies ID, and then, the results were printed (GEN III database, version 5.2.01). The results were also read in the Biolog MicroStation reader after 22 h incubation outside GEN III incubation.

## 3. Results

### 3.1. Descriptive Statistics and Survival Analysis

**Figure 2:**
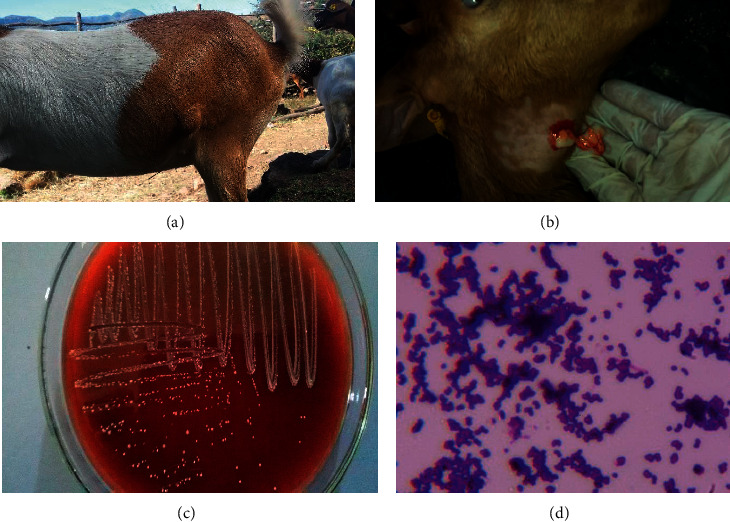
Images of CLA prescapular LN case (a), incised retropharyngeal LN case (b), sample culture on blood agar (c), and Gram stain of the bacteria (d).

During the overall eight-year clinical follow-up study period, out of 1,025 goats examined, we found 214 abscess cases clinically diagnosed as caseous lymphadenitis (Figures 2(a) and 2(b)). In the recurrent survival data analysis, there were 1, 138 observations and 214 cases (81.2% of goats with a prevalence of 18.8% were censored due to mortality, transfer to other places, or end of the study period) and the incidence rate was 0.14 cases per animal year. The prevalence was highest in Boer × CHG goats (21.03%). From a total of 214 cases, 130 have occurred once and they did not relapse, and 84 cases are twice or more relapse cases (Table 1). The incidence rate was 0.14 cases per animal year.

The most frequent cases occurred on the head region lymph node of the parotid (92 (42.99% relative percent) cases), prescapular (36 (16.82%)), and prefemoral (23 (10.75%)), and the least frequent sites were the supramammary (2.34%), cervical (1.87%), and visceral organs (liver and lung) (1.87%) (Table 2).

The typical clinical lesion is small and firm swelling of external lymph nodes of different locations (Figure 2(a)). The abscess is enclosed in a well-circumscribed fibrous tissue and when incised thick and often dry, greyish or white, purulent exudates. Cut section of some of the affected lymph nodes revealed a characteristic onion-like appearance (Figure 2(b)).

**Figure 3:**
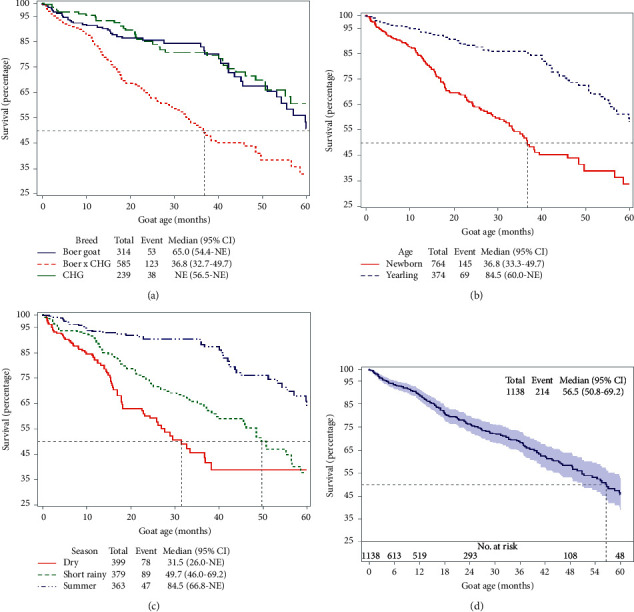
Kaplan–Meier survival function curve of caseous lymphadenitis cases from birth or entry to farm to 60 months of the follow-up period.

The unadjusted Kaplan–Meier survival function curve from birth or entry to farm to 60 months of the follow-up period of caseous lymphadenitis stratified based on the breed of goat, season, and age when the goat joined the farm indicates that the failure rate is steady and the median survival time is around 56.5 months (4.6 years) (Figure 3(d)). There was a significant difference between breed, season, and age (Figure 3). Newborn kid starts clinical cases by around 6 months of age and the failure rate is steady reaching their median survival time at 36.8 months (nearly 3 years old) (Figure 3(b)). At the end of the study, the cumulative failure rates were 68% for newborn and 64% for yearlings. The hazard is higher for newborn goats starting from first year interval up to the end while the hazard is relatively lower in the first three years for yearling goats and higher after three years of age (Table 3). The survival rate is poor for Boer cross with local Central Highland Goat and goats joined the farm during the dry season (Figures 3(a) and 3(c)).

Proportional hazard regression analysis of risk factors using the robust variance analysis of recurrent events and year as a stratifying variable showed that age, sex, and breed were associated with (*p* value < 0.05) caseous lymphadenitis occurrence (survival) and season of the year when the goat joined the farm was not associated with caseous lymphadenitis survival. Newborn goats compared with yearling age goats have 4.801 times higher probability of being infected with caseous lymphadenitis (*p* value < 0.0001). Female goats have 1.856 times higher rate of being infected compared with male goats (*p* value = 0.0023). Pure Boer goat breeds have 4.217 times higher rate of being infected (*p* value < 0.0001), and Boer cross with Central Highland Goat breeds have 4.562 times higher rate of being infected (*p* value  < 0.0001) compared with local Central Highland Goat Breed goats (Table 4).

### 3.2. Bacteriological and Biolog MicroStation with GEN III Microplate System Results

From 32 pus samples of CLA suspected goats, we were able to recover pure colonies of bacteria (Figure 2(c)). All of the samples were found to be positive for *C. pseudotuberculosis* based on cultural examination. All isolates of *C. pseudotuberculosis* were Gram-positive pleomorphic rod (Figure 2(d)), catalase-positive, urease-positive, antagonistic haemolysis with *Staphylococcus aureus* for phospholipase D (PLD) production, and nitrate-negative [4]. Out of 12 bacterial cultures tested using Biolog MicroStation with GEN III microplate system results, 10 were *C. pseudotuberculosis* and 2 of them were *C. ulcerans*.

## 4. Discussions

### 4.1. Descriptive Statistics and Survival Analysis

The present study shows the prevalence of CLA as 18.8% and the incidence rate as 0.14 cases per animal year. The cumulative failure rates were 68% for newborn aged goats and 64% for yearling aged goats. This result indicates a high rate of CLA infection rate at Ataye goat farm. The cases were characterized by poor response to local and systemic antibiotic treatments. The farm veterinarians cost a considerable time to treat the frequent cases including the recurrent abscess following incision, local treatment by iodine and savlon, and systemic antibiotic treatments using long-acting oxytetracycline.

The current prevalence is in line with reports abroad, 19.23% prevalence by Al-Gaabary et al. [21], 26% prevalence by Paton [22], and in Ethiopia 15% by Fikre and Abraha [10]. The current port is relatively higher than most reports in Ethiopia 4.7% by Molla [12], 11.7 by Abebe and Sisay [23], and 10% by Hunduma et al. [11]. The variations in the disease frequency between different studies may be attributed to the differences in the management systems and climatic conditions in each study where the viability of the causative organism in the contaminated environment is greatly affected by ambient temperature, and it may also be attributed to the endemic nature of the disease which leads to a variation in animal immunity and the degree of animal susceptibility [21]. In Australia, the prevalence of CLA had decreased from over 50% in the 1770s to approximately 20% in the late 1990s through the use of recommended CLA vaccine [22, 24]. The higher rate of infection in our study was due to semi-intensive management of goats, a higher density of flocks on the smaller grazing land, higher density at the barn, and the lower quarantine practice of clinical cases in the farm where all of them aggravates the transmission rare of the disease. There is also no vaccine available in the country.

The most affected superficial lymph node was the parotid lymph node. Similar results were reported in [2, 10]. These results may be attributed to the habit of goats that tend to scratch their shoulders and heads against walls and fences or any hard objects, resulting in a high percentage of superficial parotid, mandibular, cervical, and prefemoral lymph node infection because it drains the shoulder region [10].

The hazard of CLA was higher (*p* < 0.001) in the newborn than in the yearling age group. This result contradicts from other findings [10, 21], where age groups below 1 year are at a lower risk. This may be due to variation in the type of study and age classification method. In this result, new cases are started at around 6 months of age. The higher incidence rate in the newborn is due to the poor immunity of kids.

Female goats were at higher (*p* < 0.0023) risk of infection than male goats. Similar reports indicated a higher prevalence of CLA in female groups [21, 25]. These results may be attributed to the fact that does usually reared for older ages than bucks, as well as a relatively higher number of does usually reared in one group resulting in a high rate of contact. However, a fewer number of males are kept together mainly for the mating purpose of the farm [14, 15].

Breed variation was the other important risk factor in the proportional hazard analysis. Boer and Boer cross with local Central Highland Goats were at higher risk (*p* < 0.0001) compared with the local Central Highland Goat breeds. Generally, the imported Boer goats are at higher risk to various disease conditions [26]. This may be due to the higher resistance of local breeds due to adaptation [12].

### 4.2. Bacteriological and Biolog MicroStation with GEN III Microplate System Results

We recovered 32 *C. pseudotuberculosis* bacteria from all 32 cultured abscess samples. From 12 pure culture samples tested using the Biolog-based test, 10 of them were *C. pseudotuberculosis* and 2 of them were *C. ulcerans* positive. However, all of the 12 samples were *C. pseudotuberculosis* positive based on the biochemical test method. The Biolog-based test screened the 2 samples as *C. ulcerans*. The current result is in line with most previous findings. *C. pseudotuberculosis* were the most frequent isolated bacteria of clinical CLA abscess [10, 21, 23, 25, 27]. The present result of 2 *C. ulcerans* based on the Biolog test is the first report in Ethiopia. Although the differentiation between *C. ulcerans* and *C. pseudotuberculosis* was based on a single biochemical reaction, the trehalose test, the present results are in agreement with previous studies [28] and indicate that the microorganism isolated in the present study was *C. ulcerans*. There are also reports indicating that *C. ulcerans* was isolated from clinical cases of goat [29]. *C. ulcerans* is an emerging zoonotic disease that causes diphtheria like illness in humans [30, 31].

## 5. Conclusion and Recommendations

Clinical and bacteriological study of the caseous lymphadenitis indicates that the high prevalence of caseous lymphadenitis in this goat farm. Moreover, repeated abscess occurs after treatment. Laboratory work reveals that *C*. *pseudotuberculosis* and *C*. *ulcerans* were isolated bacteria that cause abscess disease. Thus, the frequent abscess after treatment indicates poor response of the disease to different antibiotics and antiseptic chemicals. Control and prevention in sheep and goat farms should focus on quarantine and culling of positive animals and vaccination with the CLA vaccine.

## Figures and Tables

**Table 1:** Summary of caseous lymphadenitis cases with order of recurrence.

	Caseous lymphadenitis cases							
Breed	*N*	Positive (%)	1^st^ case	2^nd^ case	3^rd^ case	4^th^ case	5^th^ case	6^th^ case
Boer goat	314	53 (16.88)	34	12	4	2	1	0
Boer × CHG	585	123 (21.03)	72	28	11	7	4	1
CHG	239	38 (15.9)	24	9	3	2	0	0
Total	1138	214 (18.80)	130	49	18	11	5	1

*Note*. CHG: Central Highland Goat.

**Table 2:** Summary of caseous lymphadenitis cases with lymph node location.

	Goat breed			
CLA location	Boer goat	Boer × CHG	CHG	Total (relative %)
Parotid LN	21	55	16	92 (42.99)
Prescapular LN	10	21	5	36 (16.82)
Prefemoral LN	2	13	8	23 (10.75)
Undefined LN	2	12	0	14 (6.54)
Mandibular LN	3	4	4	11 (5.14)
Pharyngeal LN	4	7	0	11 (5.14)
Multiple LNs	2	7	0	9 (4.21)
Skin area	1	2	2	5 (2.34)
Supramammary LN	2	1	2	5 (2.34)
Cervical LN	3	1	0	4 (1.87)
Visceral organs LN	3	0	1	4 (1.87)
Overall cases, *N* (%)	53/314 (16.88)	123/585 (21.03)	38/239 (15.90)	214/1138 (18.80)

*Note*. CHG: Central Highland Goat; CLA: caseous lymphadenitis; LN: lymph node.

**Table 3:** Life table of CLA cases stratified based on the age of goats.

Age group	Interval (months)		Total	Cases	Lost	Cumulative failures	Hazard	95% CI hazard	
								Lower	Upper
Newborn	0	12	764	61	439	0.11	0.010	0.007	0.012
	12	24	264	48	80	0.30	0.020	0.014	0.026
	24	36	136	21	53	0.44	0.018	0.010	0.025
	36	48	62	9	17	0.53	0.015	0.005	0.025
	48	60	36	5	22	0.62	0.019	0.002	0.035
	60	72	9	1	4	0.68	0.013	0.000	0.038
	72	84	4	0	4	0.68	0.000	.	.
Yearling	0	12	374	16	103	0.05	0.004	0.002	0.006
	12	24	255	16	82	0.12	0.007	0.003	0.010
	24	36	157	4	33	0.15	0.002	0.000	0.005
	36	48	120	15	33	0.27	0.013	0.007	0.020
	48	60	72	11	22	0.40	0.017	0.007	0.026
	60	72	39	3	13	0.46	0.008	0.000	0.017
	72	84	23	1	6	0.48	0.004	0.000	0.013
	84	96	16	3	12	0.64	0.029	0.000	0.062
	96	108	1	0	1	0.64	0.000	.	.

**Table 4:** Proportional hazard regression analysis results of explanatory variables effect on caseous lymphadenitis with robust standard errors.

Variables		Parameter estimate	Standard error	St. err ratio	HR	95% HR CI	*p* value
Age	Newborn vs. yearling	1.56880	0.29319	0.827	4.801	[2.702–8.529]	<0.0001
Sex	Female vs. male	0.61824	0.20285	1.159	1.856	[1.247–2.762]	0.0023
Breed	Boer vs. CHG	1.43907	0.22716	0.657	4.217	[2.702–6.582]	<0.0001
	Boer × CHG vs. CHG	1.51778	0.37440	0.912	4.562	[2.190–9.503]	<0.0001
Season	Summer vs. short rain	0.19403	0.29058	1.208	1.214	[0.687–2.146]	0.5043
	Dry vs. short rain	0.04796	0.17486	0.948	1.049	[0.745–1.478]	0.7839

*Note*. CI: confidence interval; St. err: standard error; HR: hazard ratio; CHG: Central Highland Goat.

## Data Availability

The datasets used to support the findings of this study are available from the corresponding author upon request.
